# Biomarkers of racism-based stress injury: A feasibility and correlation study

**DOI:** 10.1017/cts.2024.683

**Published:** 2024-12-27

**Authors:** Rachel Wangari Kimani, Jonathan N. Tobin, Steven W. Cole, Ann Campbell, Erich D. Jarvis

**Affiliations:** 1 Laboratory of Neurogenetics of Language, Rockefeller University, New York, NY, USA; 2 Rockefeller University Center for Clinical and Translational Science, New York, NY, USA; 3 Clinical Directors Network, Inc. (CDN), New York, NY, USA; 4 UCLA School of Medicine, Departments of Medicine and Psychiatry & Biobehavioral Science, Los Angeles, CA, USA

**Keywords:** Racism, health disparities, cardiovascular health, resilience, coping, mindfulness, stress, trauma, gene expression

## Abstract

**Background:**

Persistent discrimination and identity threats contribute to adverse health outcomes in minoritized groups, mediated by both structural racism and physiological stress responses.

**Objective:**

This study aims to evaluate the feasibility of recruiting African American volunteers for a pilot study of race-based stress, the acceptability of a mindfulness intervention designed to reduce racism-induced stress, and to evaluate preliminary associations between race-based stress and clinical, psychosocial, and biological measures.

**Methods:**

A convenience sample of African Americans aged 18–50 from New York City’s Tri-state area underwent assessments for racial discrimination using the Everyday Discrimination Scale (EDS) and Race-Based Traumatic Stress Symptom Scale. Mental health was evaluated using validated clinical scales measuring depression, anxiety, stress, resilience, mindfulness, resilience, sleep, interpersonal connection, and coping. Biomarkers were assessed through clinical laboratory tests, allostatic load assessment, and blood gene expression analysis.

**Results:**

Twenty participants (12 females, 8 males) completed assessments after consent. Elevated EDS scores were associated with adverse lipid profiles, including higher cholesterol/high-density lipoprotein (HDL) ratios and lower HDL levels, as well as elevated inflammatory markers (NF-kB activity) and reduced antiviral response (interferon response factor). Those with high EDS reported poorer sleep, increased substance use, and lower resilience. Mindfulness was positively associated with coping and resilience but inversely to sleep disturbance. 90% showed interest in a mindfulness intervention targeting racism-induced stress.

**Conclusions:**

This study demonstrated an association between discrimination and adverse health effects among African Americans. These findings lay the groundwork for further research to explore the efficacy of mindfulness and other interventions on populations experiencing discrimination.

## Introduction

**Figure 1.**
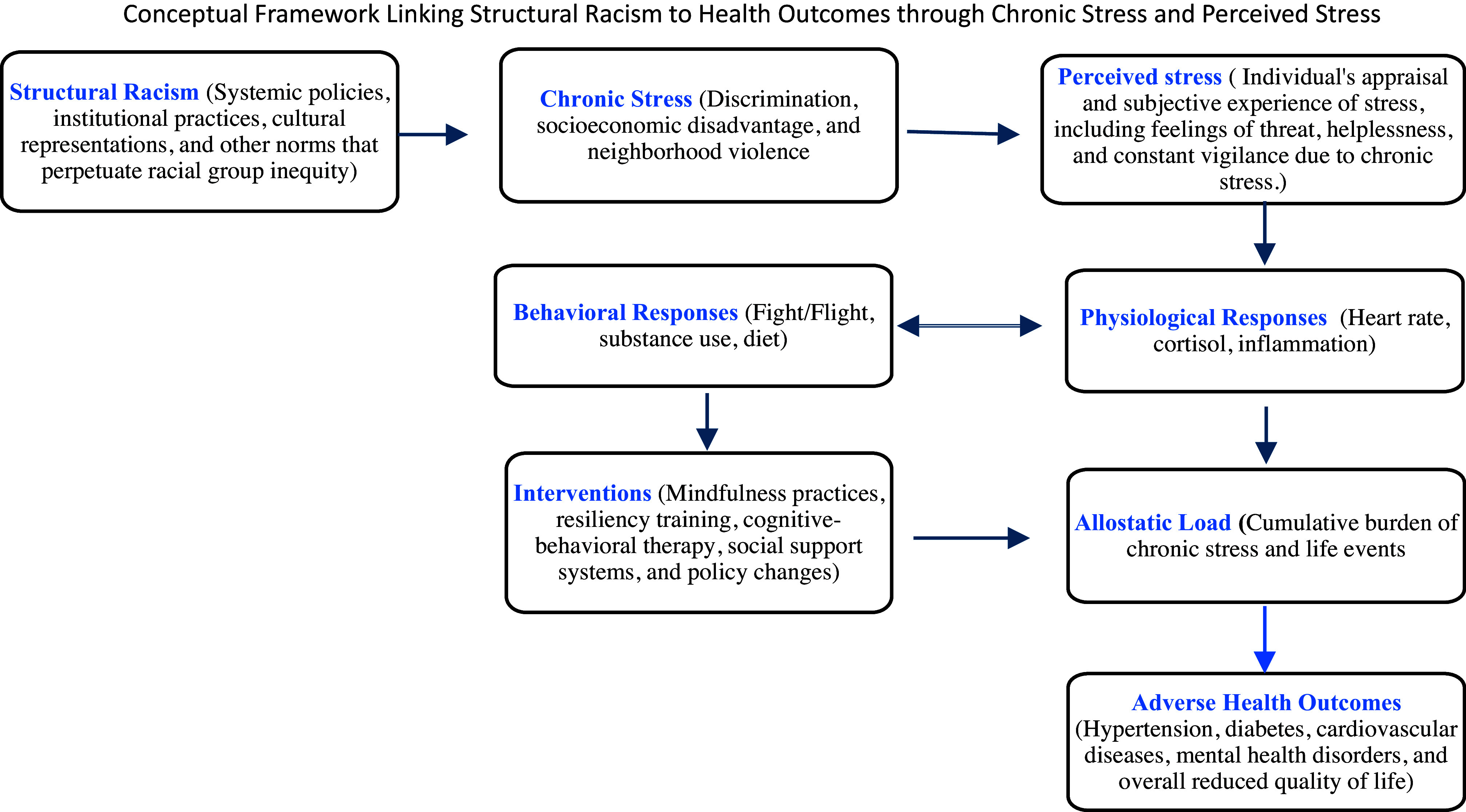
Illustrates the pathways through which structural racism contributes to adverse health outcomes in racially minoritized groups. The framework highlights the role of chronic stress and perceived stress as intermediaries that mediate the relationship between systemic inequities and health and how interventions can modify physiological and behavioral responses to reduce allostatic load and improve health outcomes.

Racially minoritized groups in the USA, including Black, Indigenous, and People of Color (BIPOC), face significant health disparities, including early disease onset, more severe disease progression, increased comorbidity and morbidity, and higher mortality rates [1]. These disparities are compounded by lower access to and poorer quality of healthcare [2–4]. The “weathering hypothesis” theorizes that such elevated health issues among minoritized groups are not only the consequences of structural racism but also physiological responses to continuous discrimination and identity threats [5]. The conceptual framework (Figure 1) below illustrates the potential pathways through which structural racism contributes to adverse health outcomes via chronic and perceived stress.

Chronic exposure to racism has been linked to neurobiological changes, specifically within cognitive regions (prefrontal cortex) and affective regions (anterior cingulate cortex, amygdala) of the brain [6–8]. Stress, in its broader sense, can be understood as a biopsychosocial response to environmental stressors[9], with psychological trauma representing a severe form of stress that can lead to neurological remodeling and dysregulation. Race-based traumatic stress, distinct in its causation from emotional pain without necessarily immediate life-threatening events, leads to varying responses based on an individual’s environmental interactions, which can manifest as symptoms like dissociation and hyperarousal [10].

Allostatic load, a term coined by McEwen[11], describes the cumulative physiological toll on the body incurred over a lifetime as it adapts to stressors. While short-term physiological responses to stress are essential for survival, the burden of chronic stress, such as that caused by chronic racism, can lead to maladaptive changes across regulatory systems, including hypothalamic–pituitary–adrenal (HPA) axis dysfunction and other physiological shifts that culminate in a state known as biological weathering [12,13]. This state is particularly pronounced in African American women, who not only face a disproportionate allostatic load but also display cellular markers of accelerated aging, such as shortened telomeres [5,14,15].

Discrimination-induced stress compounds this load, inducing a state of chronic vigilance that can cause anticipatory distress and elevate the production of allostatic mediators, thereby worsening physiological dysregulations[16–18]. Research has identified a clear link between the chronic stress of racism and a spectrum of disease biomarkers, such as increased inflammatory markers[19–21] and signs of endocrine dysregulation, further contributing to allostatic load [22,23], endocrine dysregulation[24], and neurobiological changes in the brain [6,22,25]. This confluence of adverse effects emphasizes how deeply ingrained racism is in healthcare and health disparities and highlights the need for a multifaceted approach to both understanding and addressing these issues.

Given the complex interplay between racism and stress-related health outcomes, it is evident why racism is acknowledged as a public health crisis [26]. While systemic changes are the ultimate goal for addressing the health impact of racism, there is an immediate imperative to develop effective interventions to mitigate racism-related injuries. Preliminary studies on African American women have suggested potential reductions in inflammatory markers following racism-based stress reduction programs [27].

Despite existing evidence linking racism to adverse mental health [28] and physical body outcomes [29], significant gaps remain in understanding the full extent of these effects and the potential for targeted interventions [30]. Current research has provided preliminary insights into the importance of supportive environments that foster resilience, but the lack of statistically significant findings emphasizes the need for more rigorous studies [31,32]. Additionally, perceived discrimination and psychological symptoms such as depression have been shown to have a reciprocal relationship, but this does not apply to self-rated health evaluations [28,32]. Interventions should address both to break the cycle.

This study aims to fill these gaps by creating a methodological infrastructure for future culturally responsive measures of race-based stress, tailored to African Americans, a population disproportionately affected by race-based stress. We designed a pilot study to investigate the effects of racism-induced stress on the brain and body and to assess the feasibility and effectiveness of a culturally responsive mindfulness intervention to alleviate the mental and physical impacts of this stress. We hypothesize that high levels of Race-Based Traumatic Stress Symptoms (RBTSS) will correlate positively with allostatic load and a threat-related molecular profile known as the Conserved Transcriptional Response to Adversity (CTRA) [33]. CTRA is a gene expression profile characterized by the upregulation of inflammatory genes and the downregulation of type I interferon genes, mediated by the sympathetic nervous system in response to chronic social and environmental stressors [34]. For mindfulness, we assess the feasibility to recruit participants for a study evaluating a mindfulness intervention that specifically addresses the nuances of race-induced traumatic stress. Findings from studies examining the effect of mindfulness on discrimination show an improvement in mood, blood pressure, coping, and rumination [30,35]. Interventions promoting coping flexibility and targeting ruminating thoughts have shown promise in enhancing psychological well-being and reducing symptoms of stress in diverse populations [27,36].

The specific aims of this study are:

1. To determine the feasibility of recruiting and collecting a range of psychological and behavioral variables and blood-based biomarkers of race-based stress from a sample of African Americans.
2. To assess the associations between instrumentation to measure race-based stress measures and biomarkers of stress.
3. To explore participants’ perspectives on race-based traumatic stress and examine the potential acceptability and feedback regarding a 12-week mindfulness intervention.

Ultimately, this study seeks to expand our understanding of the physiological and psychological ramifications of racism and to evaluate the potential of targeted interventions to alleviate these effects. Addressing the public health crisis represented by racism through such research is not just a scientific pursuit but a societal imperative. The study’s insights could inform interventions to reduce health disparities caused by racism and improve the well-being of minority communities.

## Materials and methods

### Participants

A convenience sample of 20 participants was recruited from advertisements on Researchmatch.org and Craigslist. All participants were screened for inclusion and exclusion requirements over the phone before scheduling their first visit. Eligibility criteria included self-identification as African American/Black, 18–50 years old, fluent in English, and born and raised in the USA.

Exclusion criteria were significant preexisting brain disease or injury, learning disability/mental retardation, current maintenance on methadone/suboxone/buprenorphine, use of illicit substances other than cannabis within the past 90 days, pregnancy, major life events in the last 30 days, and severe/chronic medical illnesses (e.g., reported HIV + status, cardiovascular disease, liver disease/cirrhosis; chronic kidney disease; current/past cancer with radiation/chemotherapy treatment). These criteria were implemented to reduce confounding variables that could amplify or mitigate neurobiological stress responses to racism-based stress [37]. For example, substance abuse could amplify or mitigate racism-based stressed. Major life events can significantly influence an individual’s stress response, thus potentially skewing the study results. Participants with a history of seizure disorders could have interactions between seizure activity, medications used to manage seizures, and the stress biomarkers measured; this exclusion ensures that the neurological conditions did not confound the study results. HIV can significantly affect the immune system and neurobiological stress responses, including the physiological impacts of HIV infection. This ensures that the study results accurately reflect the impact of racism-based stress, and not other factors, on clinical and psychological variables. We did not recruit volunteers based on whether they experienced race-based stress. The only race-based selection was that they had to be African American.

## Ethical approval

Ethical approval was obtained from the Rockefeller University Institutional Review Board (ref. 365632) and registered with ClinicalTrials.gov NCT05574933 on 10/06/2022. Funding was obtained from the National Center for Advancing Translational Sciences, National Institutes of Health, through Rockefeller University (Grant # UL1 TR001866) and the Shapiro Silverberg Foundation. All participants signed a written informed consent in English.

## Procedures

Participants completed two outpatient visits, each lasting approximately 3 hours. A licensed clinician performed a brief medical examination on the study participants. Structured Interview for DSM-5 (QUICKSCID-5) [38] was used to screen for psychiatric diagnosis. During the first visit, a point-of-care test (POCT) was used to screen for HIV. In addition, a POCT urine pregnancy test was obtained on the first and second visits among women of childbearing age.

## Measures

### Sociodemographic and clinical measures

Sociodemographic, vital, and clinical data were collected, including blood pressure measurements, height, weight, waist, and hip circumference, to calculate body mass index (BMI) and waist-to-hip ratios. Approximately 100 ml of blood was drawn for comprehensive laboratory analysis, including a complete blood count, comprehensive metabolic panel, creatinine, liver function, total and high-density lipoprotein (HDL) cholesterol, albumin, C-reactive protein (CRP), interleukin-6, tumor necrosis factor-alpha, hemoglobin A1c (HbA1c), glucose, telomere length, and gene expression profiling. Systolic and diastolic blood pressure measures were also taken. Participants also received saliva collection kits with instructions and collected three samples in 1 day: immediately upon waking, 30 minutes after, and at bedtime; the saliva cortisol awake response was measured as the difference between cortisol levels 30 minutes after waking and immediately upon waking. Allostatic load was determined using NHANES scoring [39], with clinical cutoff points that categorize risk as high (1), moderate (0.5), or low (0).

### Psychosocial assessments

Racism-based stress was assessed using the RBTSS Scale [40] and Everyday Discrimination Scale [41]. Coping with racism-based stress was measured using the Coping with Discrimination Scale (CDS) [42]. Mental health status was measured using clinical scales for resilience: the Connor-Davidson Resilience Scale (CD-RISC) [43] and trait mindfulness in daily life questionnaire (Five Facet Mindfulness Questionnaire [FFMQ]) [44]. Sleep was measured using the Pittsburg Sleep Quality Index (PSQI) [45]. Interpersonal closeness was measured using the Social Connectedness Scale [46].

### Gene expression

CTRA gene expression was measured by RNA sequencing of whole blood samples collected into PAXgene RNA tubes, as previously described [33], with cDNA libraries derived from a high-efficiency mRNA-targeted reverse transcription system (Lexogen QuantSeq 3’ FWD) and sequenced on an Illumina NextSeq instrument (Lexogen Services GmbH) acquiring an average 5.1 million 100-nt long sequencing reads per sample. Reads were mapped to the GRCh38 reference human genome (STAR aligner; average 99.7% mapping rate), with transcript abundance quantified as gene transcripts per million mapped reads. CTRA activity was measured by promoter-based bioinformatic quantification as described below [47].

### Feasibility and willingness to participate assessment

To evaluate the potential receptivity among participants for a proposed intervention targeting the alleviation of stress due to racial discrimination, we formulated an initial query related to Compassion-Based Resilience Training (CBRT). CBRT is a program that integrates mindfulness with cognitive and emotional regulation techniques and has shown promise to enhance emotional well-being, reduce markers of stress, and improve quality of life [48,49].

In preparation for a forthcoming 12-week CBRT clinical trial, the research team sought to ascertain participant interest with the following structured question:

Researchers at Rockefeller University are developing tools to reduce the impact of ongoing racism-related stress on the mind and body. One of the tools is a meditation program, which will be conducted online once a week with other AA/black people. As part of the study, we will be giving you some surveys to complete and take some bloodwork to compare if there are any changes before, during, and after completing the program. Would you be interested in participating in such a program?

### Statistical analysis

In this exploratory study, we aimed to assess whether a sample size of 20 participants would be sufficient to determine relationships between variables measured for future experimental studies and to evaluate the logistics of a proposed experiment. The primary goals were to gauge the feasibility and acceptability of the study design, as well as to explore associations among clinical, psychosocial, and gene expression data.

Given the small sample size, most of our analyses were descriptive. We conducted independent t-tests to compare continuous variables between groups, specifically gender and exposure to discrimination stress (EDS scores), presenting the results as means and standard deviations for each group. While we used a critical *p*-value of 0.05 in this preliminary study, the analysis involved 94 comparisons, which carries an increased risk of type I error (false positives). A critical *p*-value of 0.05/94 (∼0.0005) might be applied to control the study-wide type I error rate. Although we chose not to use this adjustment in this initial phase to allow for the identification of potential trends, we recognize the need for such corrections in future studies with larger datasets.

To test the feasibility of recruiting participants who meet this study’s eligibility criteria, we examined the number of people screened and enrolled during the study period. Descriptive statistics were calculated for all study variables. Using Pearson’s correlation coefficients, bivariate correlations were conducted to assess the association between race discrimination and stress biomarkers. Although *p*-values less than 0.05 were observed, these findings should be considered exploratory, given the small sample size and lack of correction for multiple comparisons. Thus, the results should be interpreted cautiously, and larger studies with appropriate statistical adjustments are required to validate these preliminary observations.

### Correlation analysis

The correlation between levels of psychological measures and quantitative clinical and biological variables were analyzed by Pearson’s correlation.

### CTRA RNA analysis

Using an established statistical analysis approach [47], a linear regression analyses was used to quantify the association between (log_2_) gene expression and race-based stress (adjusted for age and sex). Genes with a significant difference and showing > 1.5-fold differential expression in those exposed to high versus low EDS served as input into TELiS promoter-based bioinformatics analysis of NF-κB and interferon response factor (IRF) transcription factor activity, as previously described [47] (assessed by TRANSFAC position-specific weight matrices V$CREL_01 and V$IRF_Q6, along with specificity control matrices V$AP1_Q6 and V$P53_02). Statistical significance was assessed by bootstrap resampling of linear model residual vectors (controlling for correlated residuals across genes).

## Results

### Participant characteristics

A total of 34 volunteers expressed interest in the study, from which 21 were enrolled, and 20 (95%) successfully completed the study protocol. Thus, the retention rate was high (95%), demonstrating the feasibility of recruitment, assessment, and retention. The total time from the initial outreach of volunteers to the enrollment of the 21 participants was four months. Participants were assessed on their willingness to commit to the CBRT program and partake in comprehensive evaluation methods, including surveys, blood tests, and functional magnetic resonance imaging (fMRI). The majority, 18 out of 20 (90%), expressed their willingness to participate, with only 2 declining due to scheduling conflicts. All participants indicated that they were willing to complete the full battery of assessments, including undergoing fMRI, indicating a substantial willingness to participate in the intervention and its multifaceted psychosocial, laboratory, and imaging measures.

Descriptive statistics were analyzed on sociodemographic factors (Table 1). Ages ranged from 21 to 49 years, with an average of 35 (SD = 9). The gender distribution was 60% female (*n* = 12). Socioeconomic status was self-described, inclusive of a variety of economic backgrounds. Half of the participants identified as working class (50%, *n* = 10), with the remainder distributed among lower class (10%, *n* = 2), lower middle class(5%, *n* = 1), middle income (30%, *n* = 6), and upper middle class (5%, *n* = 1).

**Table 1.** Sociodemographic characteristics of participants (n = 20)

		n	%	Mean	Range	SD
Gender	Male	8	40.00%			
	Female	12	60.00%			
Age				35	28	9
	Lower class	2	10.00%			
	Working class	10	50.00%			
Social economic status	Lower middle class	1	5.00%			
	Middle income	6	30.00%			
	Upper middle class	1	5.00%			
Highest education	High school	2	10.00%			
	GED	3	15.00%			
	Some college	5	25.00%			
	Associate degree	1	5.00%			
	Bachelor degree	7	35.00%			
	Master’s degree	2	10.00%			
Religion	Christian	9	45.00%			
	Other (includes none)	11	55.00%			

Age is presented as mean with standard deviation (SD) and range. Socioeconomic status categories are self-described by participants. “Other” under religion includes (Agnostic, Muslim, Atheist).

Educational attainment was diverse, with the largest group holding bachelor’s degrees (35%, *n* = 7). Others earned an associate degree (5%, *n* = 1), achieved a master’s degree (10%, *n* = 2), or had some college education (25%, *n* = 5). The rest had completed high school or obtained a GED (25%, *n* = 5).

The religious affiliation of participants was primarily Christian (45%, *n* = 9), with the “Other” category constituting 55% (*n* = 11) of the population. This “Other” category included individuals who identified as Muslim, Agnostic, or Atheist, reflecting a range of belief systems.

### Clinical and laboratory data

The mean systolic blood pressure (SBP) was 119 mmHg (SD = 12), with females presenting a lower average SBP of 115 mmHg (SD = 11) compared to males at 124 mmHg (SD = 12) that approached significance (Table 2). The diastolic blood pressure (DBP) followed a similar pattern, with an overall mean of 73 mmHg (SD = 11), with females again registering lower at 69 mmHg (SD = 11) and compared to males at 79 mmHg (SD = 9).

**Table 2.** Gender-based differences in clinical and laboratory data

	Total = 20		Female = 12		Male = 8		
	Mean	SD	Mean	SD	Mean	SD	*p*-value
Systolic BP	119.00	12.00	115.00	11.00	124.00	12.00	0.063
Diastolic BP	73.00	11.00	69.00	11.00	79.00	9.00	0.027
Total cholesterol	175.00	25.00	181.00	26.00	165.00	22.00	0.088
HDL cholesterol	52.00	14.00	57.00	13.00	44.00	13.00	0.024
LDL	110.05	18.44	111.42	112.00	108.00	14.76	0.696
HbA1C	5.20	0.30	5.20	0.30	5.20	0.40	0.340
Waist (IN)	38.70	7.00	37.90	7.10	39.80	7.00	0.282
Hip (IN)	44.60	5.50	44.70	6.00	44.40	5.00	0.459
W/H ratio	0.86	0.09	0.85	0.09	0.89	0.08	0.130
BMI	30.84	6.48	31.26	6.89	30.21	6.21	0.367
Creatinine	1.00	0.70	1.10	0.90	1.00	0.10	0.361
Albumin	4.10	0.80	4.00	1.00	4.30	0.30	0.217
CRP	0.57	0.16	0.58	0.18	0.55	0.13	0.332
DHEA	301.00	138.00	292.00	164.00	314.00	93.00	0.370
TNF-α	0.90	0.30	1.00	0.40	0.80	0.30	0.201
IL-6	3.20	1.10	3.20	1.30	3.10	1.00	0.469
Allostatic load	2.20	1.15	2.17	1.10	2.00	1.38	0.770
Saliva cortisol_awake	0.32	0.27	0.30	0.16	0.34	0.19	0.682
Saliva cortisol_30min	0.43	0.37	0.46	0.24	0.39	0.23	0.555
Saliva cortisol_bedtime	0.15	0.12	0.17	0.16	118.00	0.07	0.414
Saliva cortisol awake response	0.11	0.21	0.15	0.22	0.05	0.18	0.311

*Note:* BP = blood pressure; HDL = high-density lipoprotein; HBA1C = glycated hemoglobin; IN = inches; W/H = waist/hip; BMI = body mass index; CRP = C-reactive protein; DHEA = dehydroepiandrosterone; TNF-A = tumor necrosis factor-α; IL-6 = interleukin-6. Allostatic load scoring using NHANES scoring[39], clinical cut points high (1), moderate (0.5), and low risk (0). Saliva cortisol awake response = cortisol_30 min – cortisol_awake .

For lipid profiles, overall total cholesterol averaged 175 mg/dL (SD = 25), while the mean HDLcholesterol was 52 mg/dL (SD = 14). A significant gender disparity was noted in HDL cholesterol, with females averaging 57 mg/dL (SD = 13) and males 44 mg/dL (SD = 13).

Other notable findings included the mean glycated hemoglobin (HbA1c) level at 5.2% for both males and females, indicating a non-prediabetic state for the participants. However, the average BMI was 30.84 (SD = 6.48) and was similar for both men and women, which would be classified as obese by the Centers for Disease Control and Prevention standards [50].

### Psychological measures

Among the psychological measures evaluated, a significant gender difference was observed in the scores of the EDS, with males reporting significantly 1.7× higher levels of discrimination (M = 19.38, SD = 8.00) compared to females (M = 11.67, SD = 6.33; *p*-value of 0.027; Table 3). No other psychometric measures, including the various factors of the Post-traumatic Growth Inventory (PTGI) and the CDS, demonstrated statistically significant differences between genders (Table 3).

**Table 3.** Gender-based differences in psychological measures and perceived discrimination

	Total *n* = 20		Female *n* = 12		Male *n* = 8		
	Mean	SD	Mean	SD	Mean	SD	*p*-value
EDS	14.75	7.86	11.67	6.33	19.38	8.00	0.027
LSC-R (overall life stress)	3.85	3.57	3.25	3.96	4.75	2.92	0.372
T_RBTSS depression (T-score)	50.00	10.00	47.51	4.81	53.73	14.44	0.179
T_RBTSS anger (T-score)	50.00	10.00	48.75	9.01	51.87	11.72	0.509
T_RBTSS physical (T-score)	50.00	10.00	47.81	6.42	53.29	13.64	0.240
T_RBTSS hypervigilance (T-score)	50.00	10.00	47.40	2.51	53.90	15.25	0.159
T_RBTSS intrusion (T-score)	50.00	10.00	47.84	4.49	53.24	14.82	0.247
T_RBTSS low self-esteem (T-score)	50.00	10.00	48.01	6.84	52.99	13.45	0.287
PTGI Factor1	11.35	11.29	13.33	11.22	8.38	11.46	0.350
PTGI Factor2	10.05	9.11	10.67	8.87	9.13	10.01	0.721
PTGI Factor3	10.90	7.81	11.67	8.19	9.75	7.59	0.605
PTGI Factor4	4.05	3.82	4.42	3.65	3.50	4.24	0.612
PTGI Factor5	8.25	5.79	9.17	5.17	6.88	6.75	0.401
CDS education/advocacy	18.20	5.92	19.42	6.04	16.38	5.60	0.271
CDS internalization	10.25	4.98	9.67	4.92	11.13	5.28	0.536
CDS drug and alcohol	10.15	3.94	8.83	2.59	12.13	4.91	0.065
CDS resistance	14.95	3.28	14.75	3.19	15.25	3.62	0.748
CDS detachment	10.90	5.17	9.17	2.33	13.50	7.15	0.064
CD-RISC	72.50	20.54	75.25	19.25	68.38	23.02	0.478
PSQI (sleep)	4.40	3.23	3.58	3.00	5.63	3.38	0.173
Social connection	90.65	16.84	92.58	17.49	87.75	16.52	0.54
(Mindfulness) FFMQ observing	9.60	3.23	9.67	3.52	9.50	2.98	0.91
(Mindfulness) FFMQ describing	11.75	3.31	12.25	3.31	11.00	3.38	0.42
(Mindfulness) FFMQ acting	11.85	2.62	12.25	2.34	11.25	3.06	0.42
(Mindfulness) FFMQ nonjudgment	12.35	3.30	13.42	3.15	10.75	3.01	0.08
(Mindfulness) FFMQ nonreactive	9.90	2.92	10.33	2.31	9.25	3.73	0.43

*Note:* EDS = Everyday Discrimination Scale; PTGI = Post-traumatic Growth Inventory; CDS = Coping with Discrimination Scale; CD-RISC = Connor-Davidson Resilience Scale; LSC-R = Life Stressor Checklist-Revised; RBTSS = Race-Based Traumatic Stress Symptoms; PSQI = Pittsburg Sleep Quality Index; FFMQ = Five Facet Mindfulness Questionnaire. T-scores are standardized scores with a mean of 50 and a standard deviation of 10, used for RBTSS subscales.

1. *Everyday Discrimination and Clinical and Laboratory Measures*

Utilizing a median EDS score of 15 as a cutoff, we divided participants into “Low EDS” and “High EDS” groups to identify potential differences among clinical and laboratory measures (Table 4). In doing so, we found the Low EDS group displayed considerably higher cholesterol HDL levels (mean = 62, SD = 9) than the High EDS group (mean = 42, SD = 12; *p* < 0.001). Additionally, the total/HDL cholesterol ratio – which is inversely related to cardiovascular health – was significantly more favorable in the Low EDS group (mean = 2.99, SD = 0.38) compared to the High EDS group (mean = 4.20, SD = 1.24; *p* = 0.009). Waist circumference, an indicator of central adiposity, also differed significantly between the groups, with the Low EDS group exhibiting a smaller mean waist size (mean = 35.6, SD = 5.5) versus the High EDS group (mean = 41.8, SD = 7.1; *p* = 0.041). Other parameters, such as blood pressure, LDL cholesterol, HbA1c, and cortisol levels, showed no statistically significant differences between the Low EDS and High EDS groups (Table 4).

**Table 4.** Comparison of clinical and laboratory measures between low and high everyday discrimination group

	Everyday discrimination (EDS)						
	Total (*n* = 20)		Low EDS		High (EDS)		
	Mean	SD	Mean	SD	Mean	SD	*p*-value
Systolic BP	119.00	12.00	115.00	10.00	123.00	13.00	0.113
Diastolic BP	73.00	7.00	73.00	6.00	78.00	9.00	0.154
Total cholesterol	175.00	25.00	183.00	27.00	166.00	20.00	0.138
HDL cholesterol	52.00	14.00	62.00	9.00	42.00	12.00	0.001
Total/HDL ratio	3.59	1.08	2.99	0.38	4.20	1.24	0.009
LDL	110.05	18.44	108.40	25.05	111.70	15.01	0.700
HbA1c	5.20	0.30	5.10	0.30	5.30	0.40	0.408
Waist (inch)	38.70	7.00	35.60	5.50	41.80	7.10	0.041
Hip (inch)	44.60	5.50	42.60	4.00	46.70	6.20	0.094
Waist/hip ratio	0.86	0.09	0.83	0.10	0.89	0.07	0.139
BMI	30.84	6.48	28.65	5.00	33.03	7.28	0.135
Creatinine clearance	1.00	0.70	0.90	0.20	1.20	1.00	0.285
Albumin	4.10	0.80	4.30	0.30	3.90	1.10	0.309
CRP	0.57	0.16	0.60	0.20	0.54	0.12	0.429
DHEA	301	138	280.00	138	321	142	0.522
TNF	0.90	0.30	0.80	0.20	1.00	0.40	0.269
IL-6	3.20	1.10	3.10	1.30	3.20	1.00	0.821
Allostatic Load	2.20	1.20	1.80	1.10	2.40	1.20	0.274
Cortisol Awake	0.32	0.17	0.27	0.14	0.37	0.19	0.228
Cortisol *T* = 30 min	0.43	0.23	0.41	0.24	0.46	0.22	0.642
Cortisol_bedtime	0.15	0.13	0.18	0.16	0.11	0.07	0.275
Cortisol_awakening	0.11	0.21	0.13	0.25	0.09	0.16	0.643

*Note:* BP = blood pressure; HDL = high-density lipoprotein; HbA1c = glycated hemoglobin; IN = inches; BMI = body mass index; CRP = C-reactive protein; DHEA = dehydroepiandrosterone; TNF-α = tumor necrosis factor-α; IL-6 = interleukin-6. Allostatic load scoring using NHANES scoring[39], clinical cut points high (1), moderate (0.5), and low risk (0). Saliva cortisol awake response (CAR) = cortisol_30 min – cortisol_awake.

**Figure 2.**
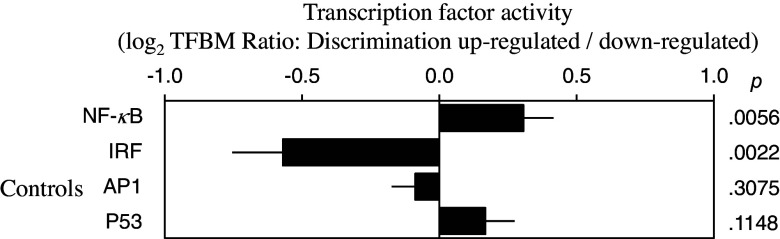
Differential activity of major pro-inflammatory (NF-kB) and antiviral (IRF) transcription control pathways in participants exposed to high (vs. low) levels of everyday discrimination, as inferred from TELiS bioinformatics analysis of genome-wide transcriptome differences. AP1 and P53 pathway activities serve as specificity controls. Data represent log2 activity ratios (high/low discrimination) ± bootstrap standard errors. IRF = interferon response factor.

RNA-Seq transcriptome profiling of blood revealed that compared to the Low EDS group, the High EDS group showed significantly greater activity (expression) of genes of the pro-inflammatory NF-kB signaling pathway (1.24-fold difference, *p* = 0.006) and lower activity of the antiviral IRF transcription control pathway (0.67-fold difference, *p* = 0.002) (Figure 2). For control analyses, we noted that the activities of the AP-1 signaling pathway and P53 tumor suppressor pathway showed no significant differences (Figure 2). These findings suggest a possible link between higher perceived everyday discrimination and several biomarkers of increased inflammation and cardiovascular risk.

### Everyday discrimination and psychometric measures

The median EDS was used to group the participants and examine correlations with psychometric measures (Table 5). The PSQI sleep score showed a mean of 4.4 for the total group. Participants with Low EDS scores had a lower mean PSQI score of 3 (SD = 1.33), and those with High EDS scores had a higher mean of 5.8 (SD = 3.99; *p* = 0.05), suggesting a poorer sleep quality with increased perceived discrimination. The mean score of the FFMQNonjudgement subscale was 12. The Low EDS group scored higher (mean = 14, SD = 2) compared to the High EDS group (mean = 11, SD = 4; *p* = 0.046). This indicates that higher levels of perceived discrimination were associated with lower levels of nonjudgmental thinking.

**Table 5.** Comparison of psychological measures between low and high everyday discrimination groups

Variables	Everyday discrimination (EDS)						
	Total *n* = 20		Low EDS		High EDS		
	Mean	SD	Mean	SD	Mean	SD	*p*-value
PSQI (sleep)	4.40	3.23	3.00	1.33	5.80	3.99	0.050
LSC-R (overall life stress)	3.85	3.57	2.30	3.65	5.40	2.88	0.049
T_RBTSS depression (T-score)	50.00	10.00	45.95	2.58	54.05	12.97	0.069
T_RBTSS anger (T-score)	50.00	10.00	45.58	4.01	54.42	12.32	0.045
T_RBTSS physical (T-score)	50.00	10.00	45.86	4.58	54.14	12.33	0.062
T_RBTSS hypervigilance (T-score)	50.00	10.00	47.35	2.69	52.65	13.72	0.247
T_RBTSS intrusion (T-score)	50.00	10.00	46.11	3.35	53.89	12.90	0.081
T_RBTSS low self-esteem (T-score)	50.00	10.00	47.08	5.16	52.92	12.87	0.199
PTGI Factor1	11.35	11.29	10.70	9.57	12.00	13.29	0.805
PTGI Factor2	10.05	9.11	8.30	8.41	11.80	9.89	0.405
PTGI Factor3	10.90	7.81	10.60	8.40	11.20	7.63	0.869
PTGI Factor4	4.05	3.82	3.80	3.65	4.30	4.16	0.778
PTGI Factor5	8.25	5.79	7.70	5.81	8.80	6.03	0.683
CDS education/advocacy	18.20	5.92	18.90	5.13	17.50	6.82	0.610
CDS internalization	10.25	4.98	9.30	4.95	11.20	5.09	0.409
CDS drug and alcohol	10.15	3.94	8.20	2.25	12.10	4.38	0.022
CDS resistance	14.95	3.28	15.00	3.65	14.90	3.07	0.948
CDS detachment	10.90	5.17	8.70	2.45	13.10	6.30	0.054
CD-RISC (resilience)	72.50	20.54	81.50	10.99	63.50	24.29	0.047
Social connect	90.65	16.84	97.30	13.34	84.00	17.96	0.076
(Mindfulness) FFMQ observing	9.60	3.23	9.50	3.75	9.70	2.83	0.894
(Mindfulness) FFMQ describing	11.75	3.31	12.90	2.23	10.60	3.89	0.123
(Mindfulness) FFMQ acting	11.85	2.62	12.50	2.32	11.20	2.86	0.279
(Mindfulness) FFMQ nonjudgment	12.35	3.30	13.80	1.99	10.90	3.78	0.046
(Mindfulness) FFMQ nonreactive	9.90	2.92	9.70	2.45	10.10	3.45	0.768

*Note:* EDS = Everyday Discrimination Scale; PTGI = Post-traumatic Growth Inventory; CDS = Coping with Discrimination Scale; CD-RISC = Connor-Davidson Resilience Scale; LSC-R = Life Stressor Checklist-Revised; RBTSS = Race-Based Traumatic Stress Symptoms; PSQI = Pittsburg Sleep Quality Index; FFMQ = Five Facet Mindfulness Questionnaire. T-scores are standardized scores with a mean of 50 and a standard deviation of 10, used for RBTSS subscales.

The CDS drug and alcohol subscale had a mean value of 10.15, with a significant difference between the Low EDS group (mean = 8.2, SD = 2.25) and the High EDS group (mean = 12.1, SD = 4.38; *p* = 0.022). The CD-RISC resilience scores also differed significantly, with the Low EDS group exhibiting higher resilience (mean = 81.5, SD = 10.99) than the High EDS group (mean = 63.5, SD = 24.29; *p* = 0.047). Similarly, the Overall Life Stressor score indicated a significant difference (*p* = 0.049), with the Low EDS group reporting fewer life stressors (mean = 2.3, SD = 3.65) compared to the High EDS group (mean = 5.4, SD = 2.88).

Additionally, the RBTSS anger subscale highlighted a statistically significant difference in reported anger levels associated with discrimination. Participants in the Low EDS group reported lower levels of anger (mean = 4, SD = 3.27), whereas those in the High EDS group reported higher levels of anger (mean = 11.2, SD = 10.04; *p* = 0.045). This difference underscores a potential link between the extent of everyday discrimination and increased feelings of anger as a component of race-based traumatic stress.

Other measures, including the FFMQ observing, describing, acting, nonreactive subscales, and the various factors of the PTGI, did not show any significant differences between the Low EDS and High EDS groups.

### Assessing interactions with sex differences

We considered whether the 1.7x higher EDS score in African American men is a confounding variable in the above analyses, which already indicates an important sex difference in EDS. Despite this sex difference, out of the 3 clinical and 6 psychological measures that differed in the EDS, only one measure, HDL cholesterol, also differed by gender. Further, the significance level for the HDL cholesterol difference by EDS is an order of magnitude higher (*p* = 0.001) than that difference by sex (*p* = 0.027). Further, the HDL difference in the EDS separation did not break down by sex. Although with a sample size of 20, it is difficult to quantify the impact of co-variates, these pilot study findings indicate that the difference for HDL is driven primarily by EDS.

### Correlation analysis

**Figure 3.**
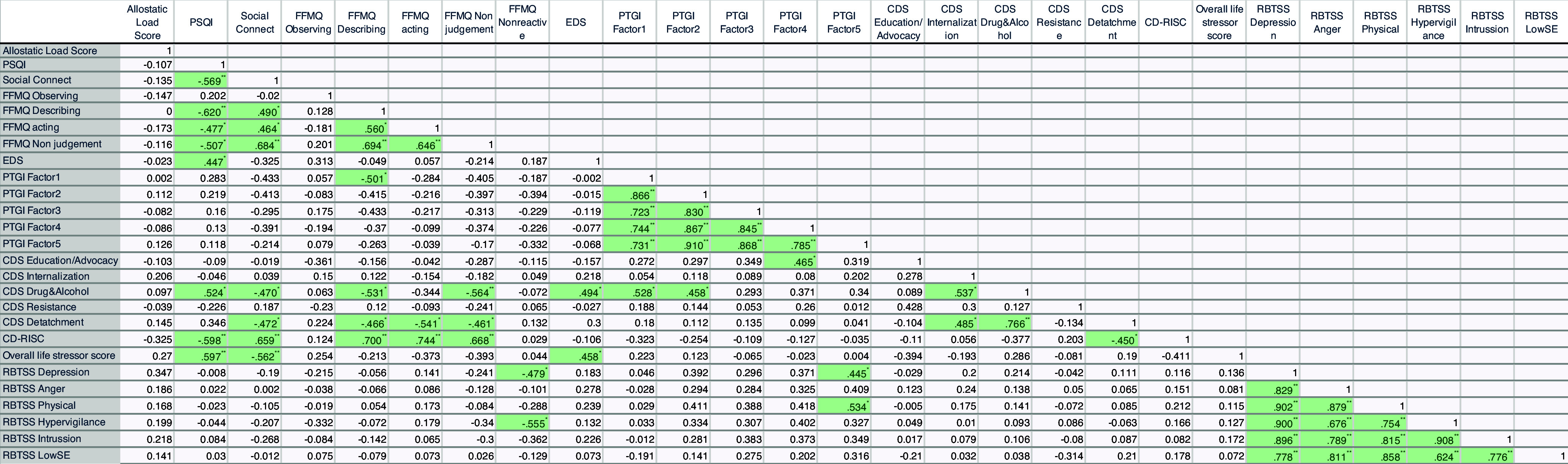
Correlation matrix – EDS = Everyday Discrimination Scale; PTGI = Post-traumatic Growth Inventory; CDS = Coping with Discrimination Scale; CD-RISC = Connor-Davidson Resilience Scale; LSC-R = Life Stressor Checklist-Revised; RBTSS = Race-Based Traumatic Stress Symptoms; PSQI = Pittsburg Sleep Quality Index; FFMQ = Five Facet Mindfulness Questionnaire. ** Correlation is significant at the 0.01 level (2-tailed). * Correlation is significant at the 0.05 level (2-tailed).

Assessing Pearson correlation coefficients (r) detailing the relationships between psychometric and biological measures within our participant cohort revealed that the PSQI sleep scores showed a strong negative correlation with Social Connection (Figure 3; r = −0.569, *p* < 0.01). Additionally, PSQI scores were negatively correlated with facets of mindfulness, such as the ability to describe (r = −0.620, *p* < 0.01), act with awareness (r = −0.477, *p* < 0.05), and nonjudgment (r = −0.507, *p* < 0.05). PSQI scores were positively correlated with the CDS drug and alcohol subscale (r = 0.524, *p* < 0.05) and Overall Life Stressor scores (r = 0.597, *p* < 0.01). A negative correlation was found between the CD-RISC resilience scores and both PSQI (r = −0.598, *p* < 0.01) and Overall Life Stressor scores (r = −0.597, *p* < 0.01). CD-RISC scores also correlated positively with measures of social connectedness and mindfulness attributes.

The RBTSS hypervigilance subscale showed a negative correlation with the nonreactive facet of mindfulness (r = −0.555 *p* < 0.05). Furthermore, EDS scores had positive correlations with CDS drug and alcohol scores (r = 0.494 *p* < 0.05) and Life Stressor scores (r = 0.458 *p* < 0.05).

## Discussion

This pilot study provides critical preliminary insights into the complex factors affecting the health of African Americans facing discrimination. Our analyses demonstrated three main findings: first, perceived racism was associated with unfavorable levels of biomarkers of health and psychological distress among African Americans; second, gender moderates the association between perceived discrimination and psychological distress in African Americans, with the association being stronger for males than for females; and third, there is an intricate interrelationship among sleep quality, mindfulness, resilience, and coping strategies in the context of everyday discrimination and stress. In addition, a majority (90%) expressed their willingness to participate in all aspects of the study, indicating the acceptability of the intervention and its associated measures. The results highlight the potential to recruit participants with variable levels of perceived racism and offer crucial methodological insights into clinical and psychosocial measures for assessing intervention studies aimed at reducing stress related to racism.

Our study corroborates a significant body of research indicating the adverse effects of perceived racism on health, linking it to detrimental biomarkers and increased psychological distress among African Americans. These findings align well with the literature that identifies racial discrimination as a chronic stressor impacting cardiovascular health, as evidenced by our examination of blood pressure and cholesterol levels [51,52] and CTRA gene expression profiles [19–21]. It also provides empirical support for the “weathering hypothesis” and the concept of allostatic load [11,14].

The stronger association for males than females in perceived discrimination on psychological distress aligns with other studies, such as those conducted in New York City, which have observed that males report higher levels of discrimination [53]. Despite these gender differences in the experience of discrimination, we did not observe significant variations in post-traumatic growth or coping strategies between males and females. These findings align with literature indicating that while Black women may navigate discrimination by entering service professions to minimize exposure to racism and sexism, whereas Black men often face racially motivated violence that challenges their gender identity, leading to unique gendered responses such as anger repression and racial identity strengthening [54]. Such findings highlight the complexity of gender-specific racial stress and the need for interventions that are sensitive to these varied experiences yet acknowledge the potential for similar psychological impact among genders. The pronounced effect among males shows the importance of considering gender differences when assessing the psychological consequences of discrimination and suggests the importance of gender-sensitive interventions.

Moreover, the intricate relationships identified among sleep quality, mindfulness, resilience, and coping strategies show the complex adaptive system influenced by everyday discrimination and stress. The observed correlation between poor sleep quality, diminished mindfulness, and heightened psychological distress highlights sleep’s pivotal role in mental health maintenance. Research indicates that stress can precipitate changes in the HPAaxis, including glucocorticoid receptor function, potentially leading to cortisol dysregulation and subsequent sleep disruption [55]. Therefore, it is plausible that the stress response triggered by discriminatory experiences could be a key disruptor of sleep health, emphasizing the importance of addressing stress management to improve sleep quality and overall well-being.

Resilience has been identified as a protective factor in our study, offering a promising therapeutic target in counteracting the adverse effects of discrimination and stress. This is consistent with the results of a national study conducted in the USA, which found that resilience can mitigate the impact of discrimination on mental health in the long run [56], highlighting the role of resilience in maintaining well-being amidst adversity. Such evidence bolsters the case for incorporating culturally responsive mindfulness interventions to alleviate the consequences of racism-related stress.

## Limitations

The study’s cross-sectional design provides a snapshot of the associations between perceived discrimination and health outcomes but cannot establish causality. Given our focus on feasibility and acceptability, we performed analyses without adjustments for multiple comparisons. This approach increases the potential for type I errors, suggesting that while the identified trends are intriguing, they should be interpreted cautiously. The findings provide preliminary insights that need to be substantiated through more rigorous statistical analysis in future studies with larger sample sizes and from a broader recruitment pool. With convenience sampling, a small sample size limits the generalizability of our findings to the broader African American population. We recommend additional observational studies to explore these associations further and provide more robust evidence, and if the associations hold up, then this should be followed by prospective randomized controlled trials with larger numbers of more diverse participants to validate findings and explore causal relationships.

In conclusion, this pilot study established a foundation for conducting larger, well-powered studies. It provides critical preliminary insights into the complex factors affecting the health of African Americans facing discrimination. The data gathered offer a nuanced understanding of the lived experiences of discrimination and its health implications. The study highlights multiple factors contributing to health disparities, including variations in biomarkers of stress, such as HDL cholesterol levels and waist circumference, and the psychological impact of perceived racism, such as psychological distress and sleep quality. By laying groundwork here, we highlight the potential for resilience and mindfulness-based interventions to mitigate the effects of discrimination, aiming for a comprehensive approach that enhances mental well-being and reduces racism-related stress. Moving forward, these findings encourage further exploration into behavioral health strategies, emphasizing the necessity for methodologically sound approaches to tackle these critical public health issues.
